# Editorial: Insights into food science and technology in Latin America

**DOI:** 10.3389/fnut.2023.1354709

**Published:** 2024-01-08

**Authors:** Andrea del Pilar Sánchez-Camargo, Janet Alejandra Gutierrez-Uribe, María Fernanda Silva, Sandra Regina Salvador Ferreira, Alejandro Cifuentes, Elena Ibañez

**Affiliations:** ^1^Grupo de Diseño de Productos y Procesos (GDPP), Department of Chemical and Food Engineering, Faculty of Engineering, Universidad de Los Andes, Bogotá, Colombia; ^2^Departamento de Biotecnología e Ingeniería de Alimentos, Monterrey Institute of Technology and Higher Education (ITESM), Monterrey, Mexico; ^3^Instituto De Biologia Agricola De Mendoza, Facultad De Ciencias Agrarias, Universidad Nacional De Cuyo, Mendoza, Argentina; ^4^Department of Food and Chemical Engineering, Faculty of Engineering, Federal University of Santa Catarina, Florianópolis, Brazil; ^5^Foodomics Laboratory, Institute of Food Science Research (CIAL-CSIC), Madrid, Spain

**Keywords:** sustainable development, undernourishment, obesity, healthy diet, valorization by-product

Food science and technology play a crucial role in shaping the food industry and ensuring the safety, quality, and sustainability of the food we consume. The Latin America and the Caribbean (LAC) region is renowned not only as a great producer of sustainable biomass but also for its rich culinary traditions and diverse agricultural practices (1). In the region, South America contributes 83% to the overall food production, Central America follows with 15%, and the Caribbean with 2%. Notably, Brazil, Argentina, and Mexico collectively generate 70% of the food in LAC, ensuring that there is a sufficient food supply to meet the caloric needs of their populations (2). However, the Food Agricultural Organization (FAO) Report on the Panorama of Food and Nutrition Security for 2023 indicates that LAC faces crucial challenges, primarily related to (i) undernourishment of the population, (ii) the prevalence of obesity and related chronic diseases, and (iii) the affordability of healthy diets (3). This phenomenon is identified as the LAC's food paradox, wherein despite substantial food production in LAC farms and fields, millions of people still suffer from hunger or malnutrition. These facts further underscore the urgent need to develop strategies to achieve the 17 Sustainable Development Goals (SDG) set for the year 2030, especially Goals 2 (Zero Hunger), 3 (Good Health and Well-being), and 12 (Responsible Consumption and Production), which still face numerous challenges to be accomplished in the countries of LAC.

In Latin America and the Caribbean, 11.6% of food is lost, equivalent to 220 million tons per year (2022), making solutions to this problem a priority. A step in this direction was taken by the United Nations, which in 2019 designated the date September 29 as the International Day of Awareness of Food Loss and Waste. This initiative aims to heighten awareness across society regarding the significance of this matter and to make progress toward achieving SDG 12, which focuses on ensuring sustainable consumption and production patterns (4).

On the other hand, the number of undernourished individuals has declined from 45.6 million in 2021 to 43.2 million in 2022, although it still surpasses the pre-pandemic level observed in 2020. The FAO report highlights a noteworthy reduction in moderate or severe food insecurity in the region, decreasing from 40.3 percent in 2021 to 37.5 percent in the past year—equivalent to 16.5 million fewer people (3). Despite this notable progress, the foremost challenge persists in effectively addressing malnutrition in LAC, necessitating solutions in agritech and food tech, including the valorization of food by-products, to originate within the region. The absence of nutritious and safe food has adverse effects on individuals' health and overall development, leading to diminished physical and cognitive growth in childhood, heightened risk of chronic diseases in adulthood, reduced productivity and income in adulthood, increased mortality rates, and elevated health system costs (2, 3, 5).

A critical concept that demands exploration when addressing the challenges confronting LAC is the Food Environment. This encompasses the combined physical, economic, and policy conditions influencing people's choices regarding food and beverages, as well as their nutritional status (6). The International Network for Food and Obesity/Non-communicable Diseases Research, Monitoring, and Action Support (INFORMAS) has recently presented a comprehensive framework for examining the food environment. This framework included considerations of food retail, provision, labeling, marketing/promotion, prices, composition, and trade and investment (6). All these factors bear significant relevance to the prevalence of obesity, serving as an additional metric for food insecurity and indicating challenges in accessing affordable, healthy food. As per the FAO report (3), the region witnessed a rise in the prevalence of overweight in children under 5 from 2000 to 2022 and an increase in obesity rates among adults from 2000 to 2016, surpassing global averages in both instances. Twenty-four percent of adults in the region suffer from obesity, and more than half of the women in LAC are affected by overweight or obesity. In addition, during the period between 2020 and 2022, amid the pandemic, there was a slight uptick in the prevalence of overweight among children under 5, shifting from 8.3% to 8.6%. In 2022, the prevalence of overweight in children under 5 stood at 9.7% in South America, 6.7% in Mesoamerica, and 6.6% in the Caribbean. Notably, South America experienced a more pronounced increase, Mesoamerica saw a modest rise, while the Caribbean remained stable (3).

Regarding the unaffordability of a healthy diet in LAC in 2022, 22.5% of the population cannot afford nutritious food. This percentage increases to 52% in the Caribbean, 27.8% in Mesoamerica, and 18.4% in South America. Moreover, the FAO report emphasizes that the surge in international food prices since 2020, exacerbated by the conflict in Ukraine and the aftermath of COVID-19, along with a regional increase in food inflation surpassing the overall rate, has heightened the difficulty for people to afford a nutritious diet (2). Indeed, currently, the main factors associated with food insecurity in LAC are two: Income loss and increase in food prices (2).

LAC researchers have been continuously making significant developments in scientific-technological capacities, industrial infrastructure, and fostering innovative entrepreneurs and food start-ups (5). In this Research Topic, titled “*Insights into food science and technology in Latin America*,” we are delighted to present four exceptional manuscripts that shed light on various aspects of this dynamic and ever-evolving field, which can contribute to providing a solution to the challenges previously mentioned. Very hot topics are addressed in the four manuscripts published (3 original research and 1 Review). In this sense, Ferrari et al., delved into the nutritional properties and nutraceutical potential of sixteen pecan (a versatile nut) cultivars grown in Uruguay. The research team employed a comprehensive approach, assessing parameters such as proximate composition and minerals, fatty acid profiles, phenolic compounds, tocopherols, anthocyanins, and antioxidant properties. The findings not only reveal substantial genetic variability among cultivars but also underscore the influence of specific environmental conditions in Uruguay. This insight into the pecan's nutritional nuances offers valuable information for farmers, nutritionists, and food scientists aiming to optimize the cultivation and utilization of this nutritious nut.

In the pursuit of functional foods for managing hypertriglyceridemia, a prevalent health concern in Latin America, the clinical trial developed by Ramírez-Jiménez et al.investigated the effects of a bean and oats snack bar on metabolic biomarkers among Mexican women. Noteworthy reductions in serum triglyceride levels and glucose concentrations were observed, accompanied by modest yet significant weight loss. The authors employed cutting-edge proteomic analysis, unraveling the intricate molecular pathways influenced by the functional snack bar. The findings not only underscore the potential of this dietary intervention but also pave the way for personalized approaches in managing hypertriglyceridemia and associated complications.

As mentioned, waste valorization has been one of the most important issues to investigate in Food Science and Technology in Latin America. It takes center stage in the Olt et al. study, as researchers explore the transformation of Tannat grape pomace, a by-product of the wine industry, into functional biscuits. Through meticulous formulation and sensory evaluation, the team creates a product that seamlessly blends health benefits with palatability. The study employs advanced analytical techniques, including HPLC-DAD-MS, to identify and quantify bioactive compounds present in the biscuits. The research demonstrates the preservation of phenolic compounds, even after baking, highlighting the potential of these biscuits as a source of antioxidants, antidiabetic agents, and antiobesity components. Furthermore, the study ensures the safety of the final product through rigorous microbiological and pesticides analysis, reassuring consumers of the quality and integrity of the functional biscuits.

On the other hand, drawing upon the rich heritage of medicinal plants in Latin America, the review carried out by Pérez-Muñoz et al. focused on *Eryngium spp.*, a group of plants with promising therapeutic potential. The authors meticulously dissect the pharmacological activities of these species, particularly in the context of managing metabolic syndrome and diabetes. The review explored the intricate mechanisms through which *Eryngium* bioactive components, including flavonoids, tannins, and terpenes, influence lipid metabolism, glucose regulation, and oxidative stress. Moreover, the review delves into innovative extraction techniques, such as cavitation and ultrasound-assisted methods, showcasing the intersection of traditional knowledge and modern technology. By providing a comprehensive overview of Eryngium's medicinal properties, this study offers a roadmap for further research and development of natural remedies in the realm of metabolic disorders.

In conclusion, the diverse research presented in this Research Topic exemplifies the originality and dedication of Latin American researchers in the field of food science and technology. Their work not only advances scientific knowledge but also holds immense potential for practical applications, benefiting both industry and public health. We express our gratitude to the authors for their valuable contributions, which serve as beacons guiding future research endeavors. As readers delve into the intricacies of these studies, we encourage them to appreciate the meticulousness of the research conducted and consider the profound implications of these findings. Through collaboration, innovation, and a deep-rooted commitment to scientific excellence, Latin America continues to make significant strides in shaping the future of food science and technology, ultimately enhancing the wellbeing of communities worldwide.

## Author contributions

AS-C: Writing—original draft, Writing—review & editing. JG-U: Writing—review & editing. MS: Writing—review & editing. SF: Writing—review & editing. AC: Writing—review & editing. EI: Writing—review & editing.
